# Effects of Contrast Medium and Vertebral Measurement Levels on Computed Tomography-Based Body Composition Parameters: Skeletal Muscle and Adipose Tissue Analysis

**DOI:** 10.7759/cureus.102643

**Published:** 2026-01-30

**Authors:** Nobuhiko Akamatsu, Wataru Gonoi, Shouhei Hanaoka, Shohei Inui, Mariko Kurokawa, Satoru Taguchi, Kotaro Sugawara, Haruki Kume, Osamu Abe

**Affiliations:** 1 Radiology, The University of Tokyo, Tokyo, JPN; 2 Radiology, The University of Tokyo Hospital, Tokyo, JPN; 3 Urology, The University of Tokyo, Tokyo, JPN; 4 Surgery, The University of Tokyo, Tokyo, JPN

**Keywords:** adipose tissue, automated segmentation, body composition analysis, contrast enhancement, low muscle mass, myosteatosis, prognosis, sarcopenia, sarcopenic obesity, skeletal muscle

## Abstract

Objective: This study aims to evaluate the effects of contrast medium administration and vertebral measurement level (L3 and L1) on computed tomography (CT)-derived body composition parameters and to establish conversion equations to enable the normalization and harmonization of these metrics across heterogeneous imaging protocols, which would aid in oncology prognostication or opportunistic screening.

Methods: A total of 203 dynamic contrast-enhanced CT examinations, including unenhanced (phase 0) and early arterial, late arterial, portal, and equilibrium phases (phases 1-4, respectively), were retrospectively enrolled in the study. Skeletal muscle area (SMA) and mean skeletal muscle density (MSMD), subcutaneous adipose tissue area (SATA) and mean subcutaneous adipose tissue density (MSATD), and visceral adipose tissue area (VATA) and mean visceral adipose tissue density (MVATD) were measured at the L3 and L1 levels across five phases (phases 0-4). Measurement changes among phases and levels were assessed statistically.

Results: The percentage changes in SMA, SATA, and VATA in phases 1/2/3/4 from phase 0 were as follows: L3 SMA, +1.1%/+2.1%/+2.8%/+3.5%; L1 SMA, +2.0%/+2.7%/+3.0%/+3.7%; L3 SATA, −0.4%/−1.9%/−2.9%/−4.7%; L1 SATA, −0.3%/−2.2%/−3.5%/−6.1%; L3 VATA, −7.5%/−17.8%/−20.0%/−22.2%; and L1 VATA, −8.5%/−20.1%/−22.6%/−23.7%. Differences in MSMD, MSATD, and MVATD in phases 1/2/3/4 compared with phase 0 were as follows (Hounsfield units): L3 MSMD, +2.1/+5.8/+7.8/+9.6; L1 MSMD, +2.9/+7.7/+9.1/+9.8; L3 MSATD, +1.2/+3.7/+4.8/+6.2; L1 MSATD, +1.2/+3.8/+4.7/+5.6; L3 MVATD, +1.5/+4.1/+5.2/+6.1; and L1 MVATD, +1.9/+4.9/+5.8/+6.3. Values between L3 and L1 showed a strong linear correlation (coefficient of determination, 0.950-0.999), suggesting interchangeability.

Conclusion: The effects of contrast phase and measurement level on CT body composition parameters were comprehensively characterized. These findings provide robust conversion equations that enable normalization of data from cohorts with mixed imaging protocols, thereby strengthening the prognostic value of body composition analysis.

## Introduction

Previous studies have revealed that sarcopenia, characterized by changes in the quality and quantity of skeletal muscle, subcutaneous adipose tissue, and visceral adipose tissue, is associated with treatment outcomes and prognosis in various diseases.

A decrease in skeletal muscle mass has been associated with a higher incidence of falls and fractures, as well as poor outcomes in various conditions, including pneumonia, cardiovascular catheterization, and pancreatitis [1-5]. It is also linked to poor prognosis in various cancers of the head and neck, lung, gastrointestinal tract, liver, bile duct, pancreas, and urinary tract [1-8]. For quantitative measurement of skeletal muscle mass, computed tomography (CT) is frequently used as a simple and minimally invasive method [9]. In particular, the skeletal muscle area (SMA) measured at the L3 vertebral level highly correlates with total body skeletal muscle mass. Shen et al. reported a correlation coefficient of 0.924 between SMA at the L3 level and total body skeletal muscle mass [10]. An L3-level skeletal muscle index (SMI, calculated as SMA divided by the square of height) is the most predominant indicator of total body skeletal muscle mass that accounts for individual physique [8].

In contrast, myosteatosis, in which adipose tissue accumulates within skeletal muscle and reduces muscle quality, has been reported to be associated with all-cause mortality in health check-up participants and with the prognosis of multiple cancers [11,12]. Skeletal muscle density, as measured by CT value, has been reported to reflect the amount of adipose tissue within the muscle [11].

Increased visceral adipose tissue mass is associated with dyslipidemia, insulin resistance, low-grade inflammation, and poor outcomes in inflammatory bowel disease [13-15]. Furthermore, associations between visceral and subcutaneous adipose tissue amounts and the prognosis of multiple cancers have been reported [13,14,16]. It has been reported that the adipose tissue area on a transaxial image at a lumbar vertebral level strongly correlates with total body adipose tissue mass [10]. Lumbar-level visceral and subcutaneous adipose tissue indices, defined as areas divided by the square of height to compensate for physical differences, are major representative indicators of total body visceral and subcutaneous adipose tissue mass [10,16,17].

Evidence has accumulated supporting the usefulness of measuring body composition parameters, including areas or densities of skeletal muscle and subcutaneous or visceral adipose tissue, with various clinically meaningful cutoff criteria proposed for these parameters. However, the methodologies for their measurement still require further elaboration and refinement. These tissues are usually contoured on CT images using established specific CT value thresholds. The limitation is that most previous studies have not considered the effects of contrast medium administration and post-contrast timing on measured CT values and calculated parameters.

As the CT values of muscle and adipose tissues increase after contrast agent administration and across post-contrast phases [18], measurements may also change when established CT value thresholds are applied. Some previous studies, with limited sample sizes, contrast conditions, and measured parameters, have reported minimal changes in SMA and subcutaneous adipose tissue area (SATA) due to contrast agent administration, whereas changes in visceral adipose tissue area (VATA) were relatively significant [19-24].

Another limitation is that the L3 vertebral level is not always included in upper abdominal CT scans and is absent in chest CT scans. In contrast, the L1 vertebral level is consistently within the imaging range of both modalities. While the L3 level is the standard for body composition analysis, the availability of L1-derived metrics could be valuable when L3 imaging is unavailable. Previous studies have also suggested the usefulness of L1-based body composition parameters [25,26].

To address these issues of methodological variability, this study was designed to comprehensively evaluate the effects of multiphase contrast administration and vertebral measurement level (L3 and L1) on CT-derived body composition parameters. The primary goal was to establish robust conversion equations that would allow normalization and interchange of these metrics, thereby providing a practical framework to harmonize data from heterogeneous imaging protocols and enhance their value as prognostic factors, which would aid in oncology prognostication or opportunistic screening.

## Materials and methods

Ethics

This study was approved by the Ethics Committee/Clinical Research Review Board of our university hospital (Approval Number 2561). As this was a retrospective study without intervention, it was determined that obtaining patient consent and registration in a clinical research registry were not necessary. This article was previously posted to the medRxiv preprint server on December 5, 2024.

Study population

This retrospective study initially enrolled 338 consecutive upper abdominal dynamic contrast-enhanced CT examinations performed using a five-phase contrast-enhanced protocol designated for hepatobiliary diseases at our university hospital between April 4, 2022, and May 19, 2022. We excluded duplicate examinations of the same patient (n=16), examinations lacking the level of the first and third lumbar vertebrae (L3 and L1 levels) or any of the five phases (n=74), examinations with skeletal muscle deformities, metal implants, or other medical devices related to prior operations (n=8), and examinations with severe edema or emaciation in which skeletal muscles were difficult to contour (n=37). The remaining 203 examinations were included in the final analysis.

CT protocol

All CT scans were performed using the following scanners: Aquilion ONE (Canon Medical Systems, Tochigi, Japan), Aquilion PRIME (Canon Medical Systems), and Discovery 750 (GE Healthcare, Chicago, IL, USA). Scanning parameters were as follows for all scanners: tube voltage 120 kVp and tube current determined using automatic exposure control. All scans were acquired during an end-expiratory breath hold. For image reconstruction, filtered back projection was used for Aquilion ONE and Aquilion PRIME, whereas Adaptive Statistical Iterative Reconstruction was used for Discovery 750. The administered dose of contrast agent was standardized to the lesser of 600 mgI/kg or 100 mL. The contrast agent used was one of the following: Iopamiron® (iopamidol; Bracco Imaging S.p.A., Milan, Italy), Iomeron® (iomeprol; Bracco Imaging S.p.A., Milan, Italy), Optiray® (ioversol; Guerbet, Villepinte, France), or Omnipaque™ (iohexol; GE Healthcare, Chicago, IL, USA). Imaging phases were defined as follows: noncontrast (phase 0); early arterial phase (phase 1, with prescan breath-hold instructions provided when the abdominal aorta at the level of the diaphragm reached 200 HU); late arterial phase (phase 2, 15 seconds after phase 1); portal venous phase (phase 3, 70 seconds after the start of contrast administration); and equilibrium phase (phase 4, 180 seconds after the start of contrast administration), for a total of five phases. The injection rate ranged from 2.2 to 3.3 mL/s. This five-phase dynamic CT protocol was selected because it includes the greatest number of phases among our registered and frequently used contrast-enhanced CT protocols, making it representative of various postcontrast acquisitions. In addition, the L1 and L3 vertebral levels were always included within the scan range. Transaxial images with a slice thickness of five mm were used for analysis.

Image analysis

**Figure 1:**
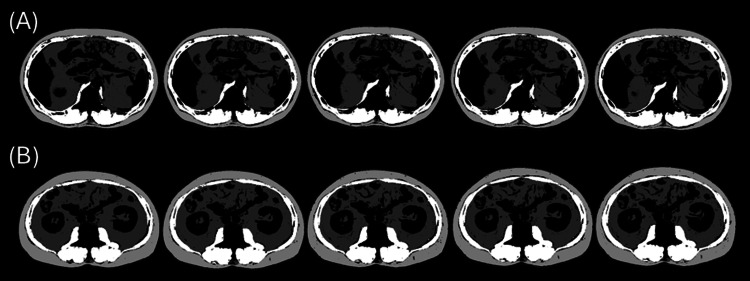
An example of body parameter segmentation analysis results from the deep-learning program Rows A and B display CT images at the L1 and L3 levels, respectively. Within each row, the images from left to right represent sequential segmentation results for the unenhanced (Phase 0), early arterial (Phase 1), late arterial (Phase 2), portal (Phase 3), and equilibrium (Phase 4) phases. White, light gray, and dark gray areas denote skeletal muscle, subcutaneous adipose tissue, and visceral adipose tissue, respectively.

Deep learning-based custom software implemented on the CIRCUS version 1.6.0 platform (http://circus-project.net), a computer-aided diagnosis tool used in previous studies [7,8,27], was applied for image analysis. For each CT examination, including five imaging phases, two transaxial planes at the midlevels of the first and third lumbar vertebrae (L1 and L3 levels), for a total of 10 images per examination, were automatically selected using the software. The software then automatically segmented all selected images into skeletal muscle, subcutaneous adipose tissue, visceral adipose tissue, and other tissues using thresholds previously proposed for noncontrast CT, as follows: skeletal muscle, −29 to 150 HU; subcutaneous adipose tissue, −190 to −30 HU; and visceral adipose tissue, −150 to −50 HU [16,17,28,29] (Figure 1). Subcutaneous adipose tissue area was defined as the region between the skin and the outer contour of the skeletal muscles that met the specified threshold. Visceral adipose tissue area was defined as the region inside the skeletal muscles that met the threshold. Board-certified radiologists (W.G., N.A., S.I.) carefully reviewed all selected slices and segmented images and manually corrected the software output as needed. At this stage, it was confirmed that no parenchymal organs were included within the regions of interest for skeletal muscle or adipose tissue and that lesions within these organs were therefore excluded from the measurements. Using the segmented images, the following six body composition parameters were extracted for each image: SMA (cm²), SATA (cm²), VATA (cm²), mean skeletal muscle density (MSMD, HU), mean subcutaneous adipose tissue density (MSATD, HU), and mean visceral adipose tissue density (MVATD, HU).

Statistical analysis

For each indicator (SMA, SATA, VATA, MSMD, MSATD, and MVATD) at the L3 and L1 levels, changes from phase 0 to phases 1/2/3/4 were calculated, respectively. The trend of these changes over imaging phases was tested using the Wilcoxon signed-rank test. Scatter plots with regression lines, coefficient of determination, and Spearman's correlation coefficients were employed to test the correlation of the indicators between the L3 and L1 levels. Statistical analyses were performed using EZR ver.1.61 (Jichi Medical University Saitama Medical Center, Tochigi, Japan), a modified statistical software derived from R Commander (The R Foundation for Statistical Computing, Vienna, Austria).

## Results

The backgrounds of the 203 cases included in the analysis were as follows: age, median 69 (interquartile range, 57-78); male/female, 132/71; and body weight, median 62 (interquartile range, 53-68).

**Figure 2:**
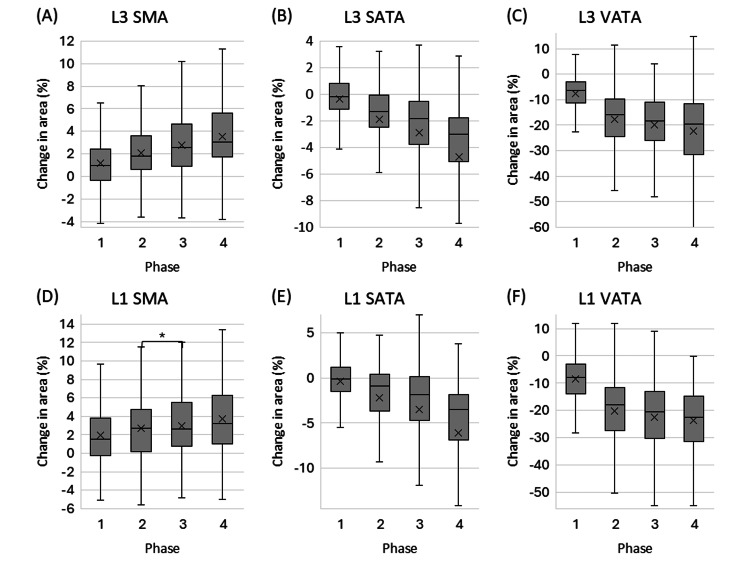
Box plots showing the percentage change in tissue area from the unenhanced phase (Phase 0) across contrast Phases 1-4. The plots display changes for: (A) L3 skeletal muscle area (SMA), (B) L3 subcutaneous adipose tissue area (SATA), (C) L3 visceral adipose tissue area (VATA), (D) L1 SMA, (E) L1 SATA, and (F) L1 VATA. The 'x' mark represents the mean value. The asterisk (*) indicates a statistically insignificant difference (p = 0.063); all other paired comparisons were statistically significant (p < 0.001).

Figure 2 depicts the change ratios of contrast phases 1/2/3/4 (early arterial/late arterial/portal/equilibrium phases) relative to phase 0 (unenhanced) for SMA, SATA, and VATA at the L3 and L1 levels. Table 1 summarizes the relative changes in SMA, SATA, and VATA for each contrast phase, benchmarked against the unenhanced phase (phase 0) as 100. In summary, SMA showed a monotonic and slight increase across phases, whereas SATA and VATA showed a slight but significant decrease across phases at both the L3 and L1 vertebral levels. The Wilcoxon signed-rank test demonstrated significant differences in SMA, SATA, and VATA among phases 0/1/2/3/4 at each level. In addition, the Wilcoxon signed-rank test revealed significant differences between most pairs of imaging phases for SMA, SATA, and VATA at both the L3 and L1 levels.

**Table 1:** Relative Tissue Area by Contrast Phase (Unenhanced = 100) Phases are defined as follows: Phase 0, unenhanced; Phase 1, early arterial phase; Phase 2, late arterial phase; Phase 3, portal venous phase; and Phase 4, equilibrium phase. SMA, skeletal muscle area; SATA, subcutaneous adipose tissue area; VATA, visceral adipose tissue area. A practical example of converting area measurements at L3 level from Phase 3 to their estimated values in Phase 0: SMA, 151.1 * 100/102.8 = 147.0 cm²; SATA, 204.3 * 100/97.1 = 210.4 cm²; VATA, 250.3 * 100/80 = 312.9 cm².

Parameter	Phase 0	Phase 1	Phase 2	Phase 3	Phase 4
L3 SMA	100	101.1	102.1	102.8	103.5
L1 SMA	100	102	102.7	103	103.7
L3 SATA	100	99.6	98.1	97.1	95.3
L1 SATA	100	99.7	97.8	96.5	93.9
L3 VATA	100	92.5	82.2	80	77.8
L1 VATA	100	91.5	79.9	77.4	76.3

**Figure 3:**
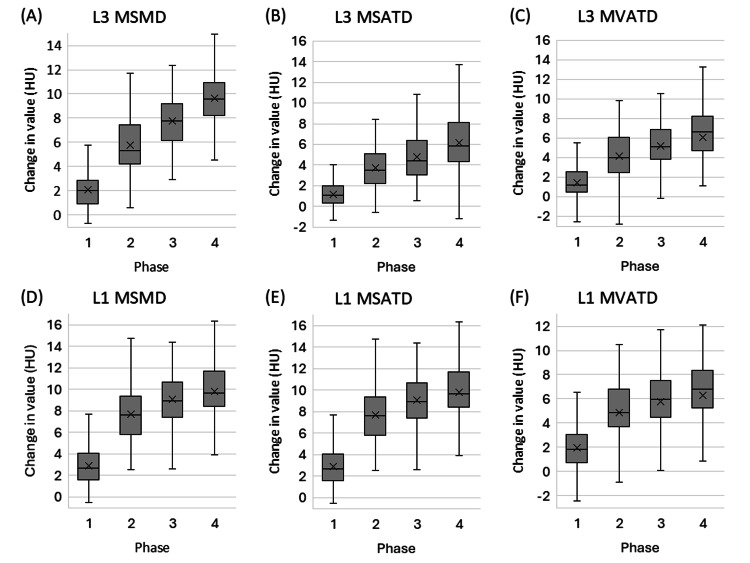
Box plots showing the change in mean tissue density (in Hounsfield Units, HU) from the unenhanced phase (Phase 0) across contrast phases 1-4. The plots display changes for: (A) L3 mean skeletal muscle density (MSMD), (B) L3 mean subcutaneous adipose tissue density (MSATD), (C) L3 mean visceral adipose tissue density (MVATD), (D) L1 MSMD, (E) L1 MSATD, and (F) L1 MVATD. The "x" mark represents the mean value. All paired comparisons of imaging phases within each parameter were statistically significant (p < 0.001).

Figure 3 shows the CT value changes in MSMD, MSATD, and MVATD at the L3 and L1 levels for phases 1/2/3/4 compared to phase 0. The absolute changes in MSMD, MSATD, and MVATD from the unenhanced phase (phase 0) are detailed in Table 2 and are presented as increases in Hounsfield Units (HU). In summary, MSMD, MSATD, and MVATD on contrast-enhanced CT showed a monotonic increase across phases at both the L3 and L1 vertebral levels. The Wilcoxon signed-rank test revealed significant differences between all ten pairs of the five imaging phases for L3 MSMD, L1 MSMD, L3 MSATD, L1 MSATD, L3 MVATD, and L1 MVATD, respectively (p < 0.001 for all).

**Table 2:** Change in Mean Tissue Density from Unenhanced Phase (in Hounsfield Units, HU) Phases are defined as follows: Phase 0, unenhanced; Phase 1, early arterial phase; Phase 2, late arterial phase; Phase 3, portal venous phase; and Phase 4, equilibrium phase. MSMD, mean skeletal muscle density; MSATD, mean subcutaneous adipose tissue density; MVATD, mean visceral adipose tissue density. A practical example of converting density measurements at L3 from Phase 3 to their estimated values in Phase 0: MSMD, 38.5 - 7.8 = 30.8 HU; MSAD, -95.6 - 4.8 = -100.4 HU; MVAD, -95.7 - 5.2 = -100.9 HU.

Parameter	Phase 0	Phase 1	Phase 2	Phase 3	Phase 4
L3 MSMD	0	2.1	5.8	7.8	9.6
L1 MSMD	0	2.9	7.7	9.1	9.8
L3 MSATD	0	1.2	3.7	4.8	6.2
L1 MSATD	0	1.2	3.8	4.7	5.6
L3 MVATD	0	1.5	4.1	5.2	6.1
L1 MVATD	0	1.9	4.9	5.8	6.3

**Figure 4:**
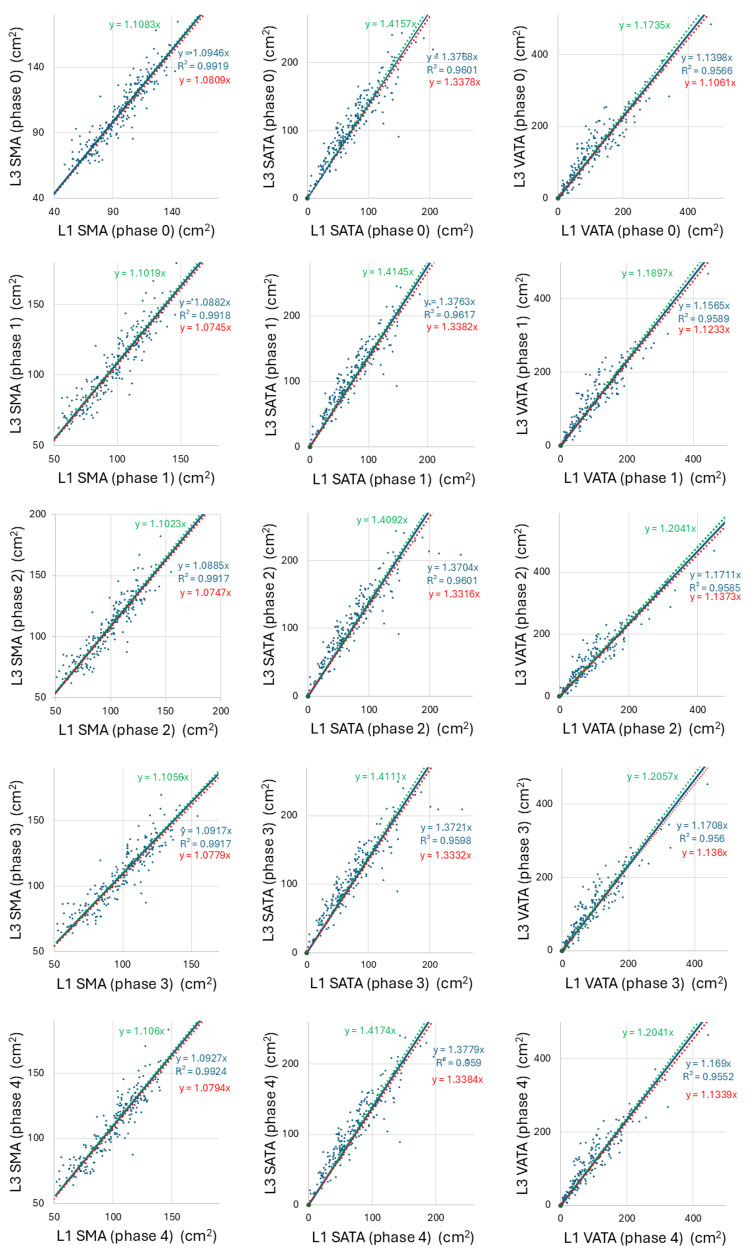
The scatter plots display a correlation between the L3 and L1 levels for skeletal muscle area (SMA), subcutaneous adipose tissue area (SATA), and visceral adipose tissue area (VATA) in unenhanced/early arterial/late arterial/portal/equilibrium phases (Phases 0/1/2/3/4) depicted along with their regression equation and coefficient of determination. The SMA, SATA, and VATA values between the L3 and L1 levels strongly correlated linearly in all imaging phases. The 95% confidence interval for the slope of the regression line is shown, with the upper and lower limits indicated in green and red, respectively.

**Figure 5:**
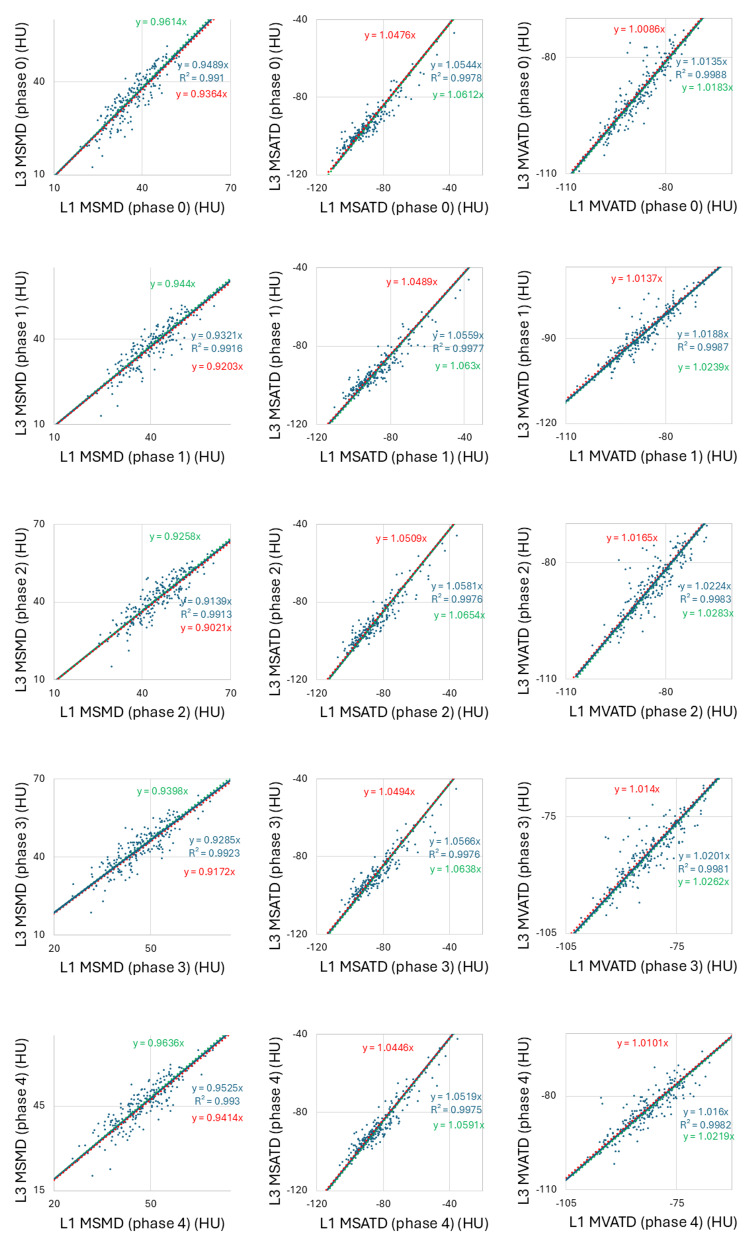
The scatter plots display a correlation between L3 and L1 levels for mean skeletal muscle density (MSMD), mean subcutaneous adipose tissue density (MSATD), and mean visceral adipose tissue density (MVATD) in unenhanced/early arterial/late arterial/portal/equilibrium phases (Phases 0/1/2/3/4) depicted along with their regression equation and coefficient of determination. The MSMD, MSATD, and MVATD values between the L3 and L1 strongly correlated linearly in all imaging phases. The 95% confidence interval for the slope of the regression line is shown, with the upper and lower limits indicated in green and red, respectively.

Scatter plots generated to test the correlation between the L3 and L1 levels for SMA, SATA, VATA, MSMD, MSATD, and MVATD in each imaging phase showed a strong linear correlation, with high coefficients of determination ranging from 0.950-0.999 across all imaging phases (Figures 4, 5). Spearman’s rank correlation coefficients ranged from 0.925 to 0.948, with p-values <0.001 for all pairs of L3 and L1 values for each body composition parameter. Measurement values between L3 and L1 can be converted interchangeably using the equations of the linear regression lines passing through the origin, as shown in Figures 4, 5.

## Discussion

In the present study, we comprehensively measured several body composition parameters on five-phase dynamic contrast-enhanced CT at the L3 and L1 levels, revealing the detailed effects of contrast medium administration and a strong correlation between L3 and L1 measurements. Furthermore, utilizing the aforementioned area change ratios and CT value changes may enable accurate estimation of measurement values at different contrast phases or vertebral levels through numerical transformation.

SMA increased monotonically following contrast agent administration over time at the L3 and L1 vertebral levels, while SATA and VATA decreased monotonically. MSMD, MSATD, and MVATD showed monotonic increases at the L3 and L1 levels after contrast agent administration. These results reflect the intravenously administered contrast agent gradually expanding its distribution from the intravascular space to the extravascular space and interstitial compartments over time [30]. While changes in SMA and SATA were minimal, VATA showed a significant decrease following contrast agent administration. This may be because visceral adipose tissue is adjacent over a wide area to parenchymal organs that exhibit more pronounced contrast enhancement compared with subcutaneous adipose tissue, and therefore VATA was underestimated due to partial volume effects.

Regarding skeletal muscle parameters, a previous study with 38 patients reported a minimal increase in SMA (+1.9%) and MSMD (+1.4 HU) in the arterial phase compared with the noncontrast phase at the L3 level [24]. Another study with 89 patients reported monotonous increases in SMA (arterial/portal/delayed phases, +0.5%/+1.5%/+1.8%) and MSMD (+6.2 HU/+11.5 HU/+14.2 HU) at the L3 level after dynamic contrast medium administration [28]. Another study with 316 healthy patients also reported increases in SMA (early/late phases, +0.8%/+1.7%) and MSMD (+5.5 HU/+8.0 HU) at the L3 level [31]. All these studies showed slight increases in SMA and MSMD over time following contrast agent administration, which was consistent with our findings.

Regarding subcutaneous and visceral adipose tissues, a previous study with 31 patients reported that abdominal to pelvic subcutaneous and visceral adipose tissue volumes decreased by up to 7.3% and 7.7% after contrast medium administration, respectively [21]. Another study reported a 9.4% decrease in SATA and a 25.4% decrease in VATA at the L1 level after contrast enhancement using an atypical CT protocol for kidney donors [32]. These results for adipose tissues were consistent with our findings.

As for inter-level comparison, in the present study, SMA, SATA, and VATA showed strong correlations between the L3 and L1 levels across all imaging phases. This suggests that SMA, SATA, and VATA at the L1 level, similar to those at the L3 level, could potentially be used for prognostic prediction in cancer patients. Regarding previous studies on correlations between different levels, a noncontrast MRI study with 155 patients with cirrhosis demonstrated a strong correlation in SMA between the L3 and L1 levels and proposed an equation relating the two levels [25]. Another noncontrast CT study with 131 patients showed strong correlations between the L3 and L1 levels for SMA and MSMD [26]. Another large-scale study with 1677 patients measured VATA and MVATD at multiple thresholds from the T10 to L4 levels [20].

The strength of the present study compared with previous investigations is its comprehensive analysis, using a larger sample size, of clinically relevant body composition parameters across multiple contrast-enhanced imaging phases and at the L3 and L1 levels. This analysis enabled the establishment of practical conversion factors and regression equations. These tools provide a validated methodology to normalize and interchange measurements obtained under varied conditions, which is crucial for harmonizing data in large-scale, multicenter cohort studies that inherently involve heterogeneous CT protocols. By enabling such standardization, our findings may improve the precision of prognostic predictions and strengthen the overall reliability of CT-based body composition analysis in diverse clinical populations.

The present study has a few limitations. The high prevalence of liver disease, homogeneous Asian ethnicity of the participants in the present cohort, and breath-hold reproducibility may be considered minor limitations.

## Conclusions

In conclusion, body composition parameters vary across different phases of contrast medium administration and measurement levels. The conversion factors provided by the present results enable more consistent body composition analyses across cohorts with mixed imaging protocols.
